# Supplementary material for: Electronic and Nuclear Quantum Effects on Proton Transfer Reactions of Guanine–Thymine (G-T) Mispairs Using Combined Quantum Mechanical/Molecular Mechanical and Machine Learning Potentials
Source: Molecules. 2024 Jun 6;29(11):2703. doi: 10.3390/molecules29112703 (PMC11173453; doi:10.3390/molecules29112703)
Supplement: Supplementary file 1 [file molecules-29-02703-s001.zip › SuppInfo.pdf]

Supplementary Materials: Electronic and Nuclear Quantum Effects on Proton Transfer Reactions of Guanine–Thymine (G-T) Mispairs Using Combined Quantum Mechanical/Molecular Mechanical and Machine Learning Potentials

Yujun Tao †, **Timothy J. Giese**‡, **and Darrin M. York**,†

1. QM/MM-ΔMLP model

While *ab initio* QM/MM simulation generally provides an accurate description for reactive processes in the condensed phase, their computational cost limits the time scale and degree of sampling that can be achieved in practice[1–4]. Semiempirical and density-functional tight-binding QM/MM simulations can be orders of magnitude more efficient, but lack the quantitative, and sometimes even qualitative accuracy afforded by their *ab initio* counterparts, especially for systems outside the chemical space for which the models were designed[1]. An alternative approach is to train machine learning potential corrections to fast, approximate semiempirical QM models so that their accuracy is comparable to that of *ab initio* QM/MM[5–10]. Early work has demonstrated that it is important to not only train the neural networks of such models against the internal QM-QM energies, but also to correct the QM/MM interaction energies out to a fixed cut-off distance[5,6]. We have developed an active learning strategy to train QM/MM-ΔMLP models to closely reproduce free energy profiles for chemical reactions in the condensed phase[5,6,11,12]. The machine learning potential correction in the current work takes the form of a short-ranged nonelectronic correction, $\Delta\text{MLP} = E_{\text{ML}}(\{\mathbf{r}\})$, where $\{\mathbf{r}\}$ is the 3N array of atomic positions. The total energy is given by

$$E_{\text{QM/MM–}\Delta\text{MLP}} = E_{\text{MM}}(\{\mathbf{r}\}) + E_{\text{QM}}(\{\mathbf{r}\};\mathbf{P}) + E_{\text{QM/MM}}(\{\mathbf{r}\};\mathbf{P}) + E_{\text{ML}}(\{\mathbf{r}\}) \quad (1)$$

E_{MM} and E_{QM} are the internal MM and QM contributions to the energy, and $E_{\text{QM/MM}}$ is the QM-MM interaction. E_{ML} is the machine learning correction (referred to as a machine learning potential correction, ΔMLP). E_{QM} and $E_{\text{QM/MM}}$ depend on the single particle density matrix, $\mathbf{P}$. In the present work, E_{QM} is calculated using the AM1/d method implemented in SQM[13], and E_{ML} is evaluated with DeePMD-kit.[14] The QM/MM-ΔMLP model described here is enabled by an interface between Sander[13] and DeePMD-kit[14] described in detail elsewhere[1]. We model E_{ML} using the Deep-Potential Range-Corrected model (DPRc) [6] that corrects the interactions between the QM atoms and their interactions with nearby MM atoms in a manner that conserves energy. In other words, the MLP correction to the QM/MM interactions smoothly approaches zero as an MM atom approaches a specified cutoff distance. Previous parametrizations of DPRc-based MLP models have used a 6 Å cutoff, which is also used in the present work. The DPRc energy is the sum of atomic contributions, E_i,

$$E_{\text{ML}} = E_{\text{DPRc}} = \sum_{i=1}^{N} E_i(\mathbf{r}_i, \{\mathbf{r}_j\}_{j\in n(i)}) \quad (2)$$

where N is the number of atoms, $\mathbf{r}_i$ is the location of atom i, and $n(i)$ denotes the set of neighboring atoms within a cutoff radius. The expressions for the E_i values from the neural network can be found elsewhere.[5,6]

2. QM/MM–ΔMLP model training

The QM/MM-ΔMLP neural network parameters were optimized to reproduce PBE0/6-31G* QM/MM energies and forces of the B-DNA system using the DP-GEN software. [15] The neural network parameters were optimized from 1 million steps using the Adam

stochastic optimization algorithm with different random number seeds [16] to yield four neural network parameter sets. Initial parameter sets were trained against 93617 samples extracted AM1/d-PhoT string calculations. The parameter sets were then updated through 7 iterations of active learning. An active learning iteration consists of 3 steps: exploration, labeling, and retraining. The exploration step performs QM/MM+ΔMLP umbrella sampling to generate new configurations. The energy and forces produced by the 4 parameter sets are compared. If the models disagree, then the sample is categorized as a "candidate". The models were deemed to disagree if the standard deviation in the MLP energy corrections was larger than 10^{-4} eV/atom or the standard deviation in the force correction exceeds 0.08 eV/Å for any atom. The "labeled" samples are a random selection of (up to) 2000 samples drawn from the pool of candidates. PBE0/6-31G* QM/MM calculations are performed on the labeled samples and included as additional reference data within the next round of training. The active learning procedure terminates when 10 % (or fewer) of the samples are selected for labeling.

Each active learning iteration sampled 1920 umbrella windows for 2 ps. 50 samples/simulation were saved for analysis. These simulations correspond to umbrella sampling describing the wGT→GT* and GT*→G*T reaction steps. The active learning procedure terminated after 7 iterations, requiring a total of 9293 *ab initio* QM/MM evaluations. Neural network parameters were produced from 4 million steps of Adam stochastic optimization with a learning rate that exponentially decays from 10^{-3} to 5×10^{-8}. [16]

Production sampling of the B-DNA tautomeric reactions was repeated 4 times with different random number seeds. The 4 trials were performed with one of neural network parameter sets (1 trial/parameter set), and the results were averaged.

3. Supporting Data Tables

Table S1. Free energy values and differences along the reaction path between classical MD and PIMD in PMF for tautomerization reactions using different QM models and methods[a]

Reactions	Method	ΔA	$\Delta A_f^\ddagger$	$\Delta A_r^\ddagger$	$e^{-\beta\Delta\Delta A}$	$e^{-\beta\Delta\Delta A_f^\ddagger}$	$e^{-\beta\Delta\Delta A_r^\ddagger}$
wGT→GT*	classical MD	3.67	21.01	17.34			
	PIMD	3.48	20.65	17.16			
	ΔΔ	−0.19	−0.36	−0.18	1.38	1.84	1.36
wGT→G*T	classical MD	3.24	21.02	17.78			
	PIMD	3.75	20.63	16.88			
	ΔΔ	0.51	−0.39	−0.90	0.42	1.93	4.57
GT*→G*T	classical MD	−0.43	7.06	7.49			
	PIMD	0.26	4.21	3.95			
	ΔΔ	0.69	−2.85	−3.54	0.31	123	395

[a] ΔΔ refers to the difference between the PIMD and classical MD results; The results were simulated with QM/MM-ΔMLP. The exponentiated free energy differences represent approximate relative populations and rates with respect to classical MD and PIMD.

References

1. Tao, Y.; Giese, T.J.; Şölen Ekesan.; Zeng, J.; Aradi, B.; Hourahine, B.; Aktulga, H.M.; Götz, A.; Merz, Jr, K.M.; York, D.M. Amber free energy tools: Interoperable software for free energy simulations using generalized quantum mechanical/molecular mechanical and machine learning potentials. *J. Chem. Phys.* **2024**.
2. McCarthy, E.; Ekesan, Ş.; Giese, T.J.; Wilson, T.J.; Deng, J.; Huang, L.; Lilley, D.M.J.; York, D.M. Catalytic mechanism and pH dependence of a methyltransferase ribozyme (MTR1) from computational enzymology. *Nucleic Acids Res.* **2023**, *51*, 4508–4518.
3. Chipot, C.; Pohorille, A., Eds. *Free Energy Calculations: Theory and Applications in Chemistry and Biology*; Vol. 86, *Springer Series in Chemical Physics*, Springer: New York, 2007.
4. Giese, T.J.; York, D.M. Quantum mechanical force fields for condensed phase molecular simulations. *J. Phys. Condens. Matter* **2017**, *29*, 383002. https://doi.org/10.1088/1361-648X/aa7c5c.

5. Giese, T.J.; Zeng, J.; Ekesan, Ş.; York, D.M. Combined QM/MM, Machine Learning Path Integral Approach to Compute Free Energy Profiles and Kinetic Isotope Effects in RNA Cleavage Reactions. *J. Chem. Theory Comput.* **2022**, *18*, 4304–4317. https://doi.org/10.1021/acs.jctc.2c00151.
6. Zeng, J.; Giese, T.J.; Ekesan, Ş.; York, D.M. Development of Range-Corrected Deep Learning Potentials for Fast, Accurate Quantum Mechanical/Molecular Mechanical Simulations of Chemical Reactions in Solution. *J. Chem. Theory Comput.* **2021**, *17*, 6993–7009.
7. Behler, J. First Principles Neural Network Potentials for Reactive Simulations of Large Molecular and Condensed Systems. *Angew. Chem. Engl.* **2017**, *56*, 12828–12840.
8. Meuwly, M. Machine Learning for Chemical Reactions. *Chem. Rev.* **2021**, *121*, 10218–10239.
9. Pan, X.; Yang, J.; Van, R.; Epifanovsky, E.; Ho, J.; Huang, J.; Pu, J.; Mei, Y.; Nam, K.; Shao, Y. Machine-Learning-Assisted Free Energy Simulation of Solution-Phase and Enzyme Reactions. *J. Chem. Theory Comput.* **2021**, *17*, 5745–5758.
10. Snyder, R.; Kim, B.; Pan, X.; Shao, Y.; Pu, J. Bridging semiempirical and *ab initio* QM/MM potentials by Gaussian process regression and its sparse variants for free energy simulation. *J. Chem. Phys.* **2023**, *159*, 054107.
11. Zeng, J.; Tao, Y.; Giese, T.J.; York, D.M. QDπ: A Quantum Deep Potential Interaction Model for Drug Discovery. *J. Chem. Theory Comput.* **2023**, *19*, 1261–1275.
12. Zeng, J.; Tao, Y.; Giese, T.J.; York, D.M. Modern semiempirical electronic structure methods and machine learning potentials for drug discovery: Conformers, tautomers, and protonation states. *J. Chem. Phys.* **2023**, *158*, 124110. https://doi.org/10.1063/5.0139281.
13. Case, D.A.; Aktulga, H.M.; Belfon, K.; Cerutti, D.S.; Cisneros, G.A.; Cruzeiro, V.W.D.; Forouzesh, N.; Giese, T.J.; Götz, A.W.; Gohlke, H.; et al. AmberTools. *J. Chem. Inf. Model.* **2023**, *63*, 6183–6191.
14. Zeng, J.; Zhang, D.; Lu, D.; Mo, P.; Li, Z.; Chen, Y.; Rynik, M.; Huang, L.; Li, Z.; Shi, S.; et al. DeePMD-kit v2: A software package for deep potential models. *J. Chem. Phys.* **2023**, *159*, 054801. https://doi.org/10.1063/5.0155600.
15. Zhang, Y.; Wang, H.; Chen, W.; Zeng, J.; Zhang, L.; Han, W.; E, W. DP-GEN: A concurrent learning platform for the generation of reliable deep learning based potential energy models. *Comput. Phys. Commun.* **2020**, *253*, 107206. https://doi.org/10.1016/j.cpc.2020.107206.
16. Kingma, D.P.; Ba, J. Adam: A Method for Stochastic Optimization **2017**.

Disclaimer/Publisher's Note: The statements, opinions and data contained in all publications are solely those of the individual author(s) and contributor(s) and not of MDPI and/or the editor(s). MDPI and/or the editor(s) disclaim responsibility for any injury to people or property resulting from any ideas, methods, instructions or products referred to in the content.
