# Supplementary figures and images for: Electronic and Nuclear Quantum Effects on Proton Transfer Reactions of Guanine–Thymine (G-T) Mispairs Using Combined Quantum Mechanical/Molecular Mechanical and Machine Learning Potentials
Source: Molecules. 2024 Jun 6;29(11):2703. doi: 10.3390/molecules29112703 (PMC11173453; doi:10.3390/molecules29112703)

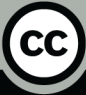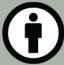

BY

Supplement: Supplementary file 1 [file molecules-29-02703-s001.zip › Definitions/logo-ccby-eps-converted-to.pdf]

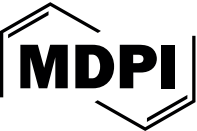

Supplement: Supplementary file 1 [file molecules-29-02703-s001.zip › Definitions/logo-mdpi-eps-converted-to.pdf]

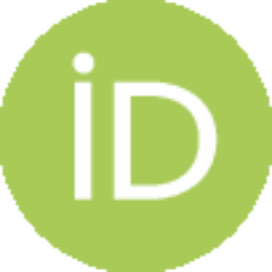

Supplement: Supplementary file 1 [file molecules-29-02703-s001.zip › Definitions/logo-orcid-eps-converted-to.pdf]

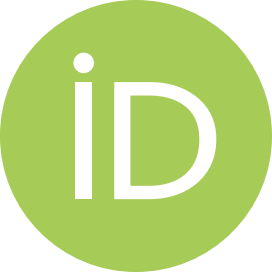

Supplement: Supplementary file 1 [file molecules-29-02703-s001.zip › Definitions/logo-orcid.pdf.orig]

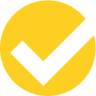

check for  
updates

Supplement: Supplementary file 1 [file molecules-29-02703-s001.zip › Definitions/logo-updates-eps-converted-to.pdf]

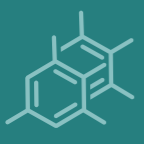

*molecules*

Supplement: Supplementary file 1 [file molecules-29-02703-s001.zip › Definitions/molecules-logo-eps-converted-to.pdf]

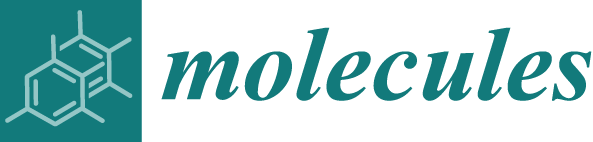

Supplement: Supplementary file 1 [file molecules-29-02703-s001.zip › Definitions/molecules-logo.png]
